# Physical Health Monitoring for Patients Within an Assertive Outreach

**DOI:** 10.1192/bjo.2025.10583

**Published:** 2025-06-20

**Authors:** Christiana Elisha-Aboh, Awais Akbar

**Affiliations:** Tees, Esk and Wear Valley Foundation Trust, York, United Kingdom

## Abstract

**Aims:** This audit highlights the well-documented disparity in life expectancy between individuals with severe mental illnesses, such as schizophrenia, and the general population, attributed in part to inadequate access to and engagement with healthcare services. Addressing this issue aligns with the Government’s commitment to achieving parity between physical and mental healthcare standards.

The audit aims to evaluate progress in physical health monitoring for patients, identify improvement areas, and assess practices against NICE and NCAP standards.

**Methods:** In January 2021, the team appointed a healthcare assistant to enhance physical health monitoring for patients with severe mental health conditions. An audit of 39 patients active between January 2023 and January 2024 reviewed electronic records, focusing on physical health results, case notes, and correspondence to assess outcomes.

**Results:** In 2021, 83% of patients had documented monitoring for HbA1c/RPG and lipids, with four documented refusals. By 2023, these rates improved to 100% (24/24) for both measures, although 15 patients refused monitoring. Family history documentation for diabetes, hypertension, and hyperlipidaemia showed significant progress, increasing from 0% in 2021 (10/10) to 100% in 2023 (25/25). For the period from January 2023 to January 2024, 100% of patients (39/39) were offered comprehensive physical health monitoring, maintaining the 2021–2022 rate. All cases documented tobacco, alcohol, and substance use clearly. Dietary and exercise advice remained consistent at 100% across both years. Notably, in 2023, 100% (3/3) of patients with diabetes and hypertension indications received intervention offers, while no patients required intervention for dyslipidaemia. Additionally, 100% of patients were offered smoking cessation and support for reducing alcohol and substance misuse, with refusals clearly documented.

**Conclusion:** The 2023 audit highlighted both challenges and progress in physical health monitoring. Although 15 patients declined HbA1c/RPG and lipid monitoring – an increase from 4 in 2021, this signals a need for improved patient engagement strategies, such as rapport-building and tailored education on monitoring benefits. Positively, documentation of family history for diabetes, hypertension, and hyperlipidaemia reached 100% in 2023, indicating effective recognition of familial risk factors. To further enhance documentation consistency, adding a dedicated family history section to the annual physical health check form is recommended. Overall, the audit shows significant progress, achieving 100% rates for key metrics and aligning with national standards. However, ongoing efforts are needed to engage patients who refuse interventions, ultimately improving outcomes and reducing health disparities among individuals with severe mental illnesses.

